# Supersaturation of VEP in Migraine without Aura Patients Treated with Topiramate: An Anatomo-Functional Biomarker of the Disease

**DOI:** 10.3390/jcm10040769

**Published:** 2021-02-15

**Authors:** Ciro De Luca, Sara Gori, Sonia Mazzucchi, Elisa Dini, Martina Cafalli, Gabriele Siciliano, Michele Papa, Filippo Baldacci

**Affiliations:** 1 Laboratory of Morphology of Neuronal Network, Department of Public Medicine, University of Campania “Luigi Vanvitelli”, 80138 Napoli, Italy; michele.papa@unicampania.it; 2 Neurology Unit, Department of Clinical and Experimental Medicine, University of Pisa, 56126 Pisa, Italy; sara.gori@ao-pisa.toscana.it (S.G.); mazzucchi.s@gmail.com (S.M.); dini.elisa87@gmail.com (E.D.); gabriele.siciliano@unipi.it (G.S.); filippo.baldacci@unipi.it (F.B.); 3 Unit of Neurorehabilitation, Department of Medical Specialties, University Hospital of Pisa, 56126 Pisa, Italy; martinacafalli@gmail.com; 4 SYSBIO Centre of Systems Biology ISBE.ITALY, University of Milano-Bicocca, 20126 Milano, Italy

**Keywords:** migraine without aura, biomarker, neurophysiology, neuroanatomy, visual cortex, VEP, migraine cycle, allodynia

## Abstract

Migraine is a primary headache with high prevalence among the general population, characterized by functional hypersensitivity to both exogenous and endogenous stimuli particularly affecting the nociceptive system. The hyperresponsivity of cortical neurons could be due to a disequilibrium in the excitatory/inhibitory signaling. This study aimed to investigate the anatomo-functional pathway from the retina to the primary visual cortex using visual evoked potentials (VEP). Contrast gain protocol was used in 15 patients diagnosed with migraine without aura (at baseline and after 3 months of topiramate therapy) and 13 controls. A saturation (S) index was assessed to monitor the response of VEP’s amplitude to contrast gain. Non-linear nor monotone growth of VEP (S < 0.95) was defined as supersaturation. A greater percentage of migraine patients (53%) relative to controls (7%) showed this characteristic. A strong inverse correlation was found between the S index and the number of days separating the registration of VEP from the next migraine attack. Moreover, allodynia measured through the Allodynia Symptoms Check-list (ASC-12) correlates with the S index both at baseline and after 3 months of topiramate treatment. Other clinical characteristics were not related to supersaturation. Topiramate therapy, although effective, did not influence electrophysiological parameters suggesting a non-intracortical nor retinal origin of the supersaturation (with possible involvement of relay cells from the lateral geniculate nucleus). In conclusion, the elaboration of visual stimuli and visual cortex activity is different in migraine patients compared to controls. More data are necessary to confirm the potential use of the S index as a biomarker for the migraine cycle (association with the pain-phase) and cortical sensitization (allodynia).

## 1. Introduction

Cortical excitability anomalies among migraine patients have been studied through the years with both electrophysiology and functional neuroimaging technologies [1,2,3,4,5,6,7]. Thalamocortical and cortical dysfunctional connectivity seems to be implicated in the pathophysiology of migraine [2,3,4,8] and its clinical counterparts (e.g., pain and cephalic and extracephalic allodynia) [9,10], although these anomalies tend to differ considering the ictal and interictal phases of migraine [11,12]. 

The study of the visual system can be easily performed from the retina to the cortex with visual evoked potentials (VEP). VEP are safe, non-invasive and can be reassessed coherently on the same patient or control. Varying the frequency of visual stimulation, the examiner could elicit cortical responses with transient (T-VEP) or steady-state (SS-VEP) waveforms. Both these VEP analyses have been used to characterize migraine patients [6,13,14], even in combination with neuroimaging [5,15]. 

Hyperresponsiveness of visual cortical areas, during intercritical phases, seems to be supported by electrophysiological experiments [5,6] and functional magnetic resonance imaging (fMRI) [16,17,18]. 

Visual cortex neurons exert their response to stimuli with a saturation curve that impedes a linear growth to the increase in contrast (i.e., contrast gain). In vivo experiments on primate neurons of V1 and V2 areas showed that excitatory signals, responding to contrast gain, are counterbalanced by intracortical inhibitory feedbacks [19,20].

In primate experimental settings, the contrast gain produces a plateau that represents the saturation of about 75% of V1 and V2 cells, while the remaining percentage progressively reduces their firing with contrast gain, rather than stabilizing it. This electrophysiological phenomenon is defined as supersaturation at high contrast, probably due to overcompensation of cerebral inhibitory networks [21,22]. 

The response to visual stimuli depends on the inhibitory/excitatory balance through the cortical network and may comprehend subcortical diencephalic relays pathways [23]. VEP allows measuring the summation of the electrical activity of the occipital region, where the visual cortex resides, in response to the presented stimulus and contrast gain. The precise electrical sources of VEP signal have not been utterly understood, nonetheless, the pivotal areas seem to be V1, V2, and V5 [24].

Through an SS-VEP study, the supersaturation was demonstrated in 43% of migraine patients and 14% of controls [6]. No studies, as far as we know, are investigating the T-VEP supersaturation in response to contrast gain, comparing migraine patients at baseline with controls and in the follow-up after prophylactic treatment. The employment of T-VEP instead of SS-VEP could be useful since they are more diffused in clinical practice. 

Our aim was to define the possible context-of-use of VEP amplitude progression in response to contrast gain as a diagnostic migraine biomarker and predictor of response to preventive treatments with topiramate. Topiramate has been largely used as preventive drug in migraine for two decades, and it could modify the excitatory/inhibitory balance of both retinal and cortical firing based on the following mechanisms of action:

(1) inhibition of α-amino-3-hydroxy-5-methyl-4-isoxazolepropionic acid (AMPA) and kainate-type glutamate receptors (AMPARs, KARs), (2) amplification of chloride currents of γ-amminobutirrico (GABA)A receptors (both cortical and retinal subtypes), (3) state-dependent block of a sodium channel, and (4) high voltage-activated calcium channels blockage [25,26,27].

## 2. Materials and Methods

### 2.1. Study Population

We recruited 15 consecutive outpatients of the Headache Centre of the University of Pisa with the following enlisted inclusion criteria (IC) and exclusion criteria (EC). 

IC: (1) age ≥ 18 years; (2) normal neurologic evaluation; (3) brain MRI examination without pathological findings; (4) absence of migraine preventive treatment for at least 3 months before study inclusion; (5) patient fulfilling the diagnostic criteria for Migraine without aura according to International Classification of Headache Disorders-3 (ICHD-3) [28]; (6) episodic migraine with a frequency of headache attacks between 5 and 10 days per month (7) ophthalmologic evaluation resulting in corrected visual acuity of 0.8 decimals or higher on standard visual acuity charts, normal anterior segment, clear dioptric elements, and intraocular pressure lower than 21 mmHg and healthy fundus oculi; (8) last migraine attack ceased at least 72 h before the first visit of the study. 

EC: (1) comorbid medical disorders and treatments for chronic systemic diseases; (2) patients with headache attacks fulfilling the criteria for migraine with aura or other primary or secondary headaches’ diagnosis, according to ICHD-3; (3) high-frequency episodic migraine (more than 10 headache attacks per month) or chronic migraine diagnosis according to ICHD-3; (4) low-frequency episodic migraine with less than 4 headache attacks per month; (5) pregnancy or breastfeeding.

The range of attack frequency for episodic migraine was determined to satisfy the recommendation of international guidelines to start a preventive treatment and to increase the chance of VEP recording in the intercritical period.

The controls were selected among healthy subjects not genetically related to the patients and hospital workers. Individuals with a significant headache history, assessed through a clinical interview, were exclude. Besides, subjects with a positive history of medical disorders or with chronic medication use were excluded from the present study.

All patients were topiramate-naïve and were not enrolled in previous clinical studies on migraine. This study has been performed in accordance with the Declaration of Helsinki, and it was approved by the local ethics committee. All patients and controls provided valid written, informed consent before study inclusion and could leave the study at any time and for any reason.

### 2.2. Clinical Evaluation

Patients underwent two visits at the interval of 111 ± 7 days (21 days of drug titration protocol and 90 days of stable treatment): baseline (T0) and follow-up (T1) evaluation. At the end of T0, topiramate was prescribed to the enrolled patients with a starting dose of 25 mg once per day, increased in steps of 25 mg every week, up to the dose of 100 mg per day (50 mg twice per day after 4 weeks).

Clinical features of migraine were measured though clinical interview, patients’ diary (years of illness, headache frequency, days to the next migraine attack, and severity of the average attack), and employing appropriate scales during T0 and T1 visits: Verbal Numeric Scale (VNS) to express the pain intensity of the average attack, Headache Impact Test (HIT-6) and Migraine Disability Assessment (MIDAS) for the valuation of headache/migraine-related disability, and Allodynia Symptoms Check-list 12 (ASC-12) for the specific measure of allodynia. Additionally, to assess comorbidities, we administered the Fatigue Severity Scale (FSS) to evaluate migraine-associated fatigue and Generalized Anxiety disorder (GAD–7) and Patient Health Questionnaire (PHQ–9) for mood disorders and anxiety. Sleep quality and chronotype were determined through the Epworth Sleepiness Scale (ESS), reduced Morningness-Eveningness Questionnaire (rMEQ), and Pittsburgh Sleep Quality Index (PSQI)

Following 3 months of stable topiramate dosage (50 mg twice per day), patients were reevaluated, the same questionnaires were administered, while drug compliance and headache frequency were recorded by the patients on a diary.

### 2.3. Electrophysiological Evaluation

VEP were registered when patients were free from headache for at least 72 h (interview-based assessment at T0). A phone-call was made 48 h after the registration to verify the occurrence of a headache attack. The intercritical period was defined by the absence of pain 72 h before and 48 h after the evaluation. Indeed, VEP acquisition could fall in the intercritical period if the patient did not report pain during the following 48 h. We defined the critical period when the days separating the registration and the attack onset were <2. This premise does not apply to the control group.

VEP recording was performed in line with the International Society for the Clinical Electrophysiology of Vision guidelines [29].

Monocular VEP were registered with a patch covering the non-recorded eye. The visual pattern stimulator was Mod. BM1502 (Biomedica Mangoni s.n.c.), the preamplifier ISA1004 EP was coupled with the amplifier BrainQuick BQPCI (Micromed S.p.A.) and the elaboration software was SystemPLUS v.1.2 (Micromed S.p.A.).

Briefly, the patient was positioned, comfortably seated, at 100 cm from a cathode ray tube (CRT) display with gamma correction. Visual stimuli were characterized by a black and white checkboard with reversal pattern. The checkboard (full-field 32°, 18′ checks, with central fixation point) had a mean luminance of 52 cd/m^2^, and a temporal frequency of 1 Hz to elicit a transient response. The recording was made using Ag/AgCl superficial electrodes positioned according to international standards of VEP montage [29], i.e., the active electrode was positioned in the O_z_ at 10% nasion-inion distance (International 10–20 system), about 3 cm above the inion, on the midline with the reference electrode positioned in F_z_ (on average 11 cm above the nasion), and the ground electrode in C_z_. Electrode impedance was balanced among electrodes and did not exceed 5 kOhms

The measure of contrast gain was obtained presenting visual stimuli of 6%, 10%, 20%, 50%, 75%, and 100% (theoretical) of Michelson contrast. The different contrasts were presented increasing progressively for each session, to avoid contrast adaptation. Furthermore, 2 min of exposure to a 0% contrast gray screen, with the same luminance, was performed among each session of recording. The averaging of 200 signals for every contrast was achieved with 25 signals at the time, to lower the discomfort of prolonged stimulation, reducing artifacts, and minimizing the potential habituation deficit reported in migraine patients [30,31]. Patients’ preparation took about 15 min, while the recording session lasted about 35 min. These settings elicit both foveal and parafoveal transient responses, which are characterized by three consistent deflections of the electric signal with alternating polarity, reproducible for both latency and morphology, i.e., the principal positive wave has a latency of approximatively 100 ms (P100) and is preceded and followed by two negative waves, the first with 70–75 ms latency (N75), and the latter with a peak at 130–145 ms (N145). VEP values were calculated considering the peak-to-baseline amplitude in microvolts of the three waves. The peaks were manually assigned, and measures were recorded by an expert physician, blinded to group assignment. Latencies were also recorded but showed no significant differences between groups.

Patients’ VEP were registered at T0 and T1, while the controls’ VEP were registered once, after the clinical interview.

### 2.4. Mathematical Model for Contrast Gain Assessment

The VEP amplitude response to contrast gain was modeled to differentiate between monotonic and non-monotonic saturation (i.e., supersaturation) with the definition of a saturation index (S):

(1) $$S=1-\;\frac{A_{max}-\;A_{100}}{A_{max}}$$

where *A_max_* represents the maximum amplitude for the examined subject and *A*_100_ represents the VEP amplitude at 100% of contrast. Ideally, the value of 1 characterizes a monotonic saturation at high contrast, while values < 1 indicate supersaturation with paradoxical reduction at high contrast. The *S* index has both a qualitative value, as aforementioned, and quantitative significance since lower values of *S* represent greater differences between the maximum amplitude recorded and the amplitude registered at 100% of contrast. To avoid confounding data due to sampling or measurement, a 5% difference between the raw data of *A_max_* and *A*_100_ (*S* = 0.95) has not been considered supersaturation. 

Furthermore, to standardize the VEP data analysis and rule out the interindividual variation of raw microvolts values, the amplitude is expressed as normalized to the 100% contrast, following the formula:

(2) $$A_{n\%}=\frac{A_{n}}{A_{100}}\;$$

where *A_n_* is the raw amplitude of VEP potentials given the *n* contrast, while *A_n_*% represents the amplitude at *n* contrast, normalized for the amplitude registered at maximum contrast in the same patient.

### 2.5. Statistical Analysis

The quantitative variables, not normally distributed, are expressed as the median, and interquartile range (IQR) categorical variables are expressed as frequency percentages. The comparison between quantitative paired variables (T0 and T1) from migraine population was performed using Wilcoxon test. Mann–Whitney U test was employed to confront unpaired quantitative variables between migraine and control groups. Spearman rank correlations were calculated to test for a relationship between S index and migraine characteristics. The categorical dichotomous variables were compared through the chi-square test with continuity correction or the Fisher exact test (FET), when appliable. 

## 3. Results

The patients enrolled in the study were 15 (73% female and 27% male), while the control group constituted of 13 subjects (69% female, 31% male) with no differences considering age (U = 101; *p* = 0.89) and gender (FET *p* = 1) as summarized in Table 1.

**Figure 1:**
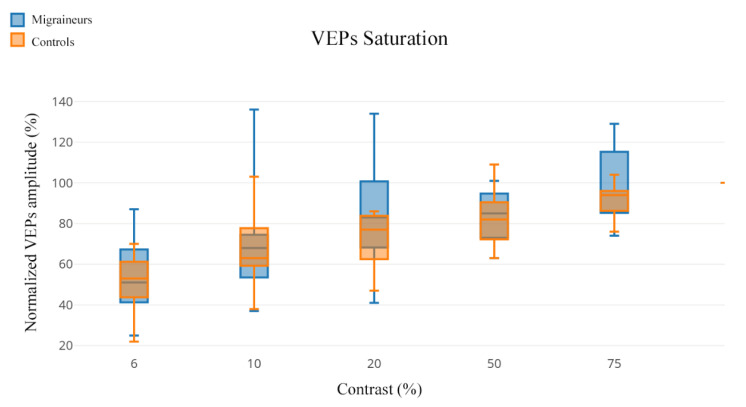
Graphic representation of normalized amplitude variations in response to contrast gain for both migraineurs and controls. The plot evidences a higher variability of visual evoked potentials (VEP) amplitude response in migraineurs, particularly at the contrast of 20%.

Figure 1 shows VEP normalized amplitude in response to contrast gain in both patients and controls. The overlap between the amplitude measured at a single contrast does not permit the distinction between the two groups. Nonetheless, greater variability was observed in migraineurs, particularly at the contrast of 20%. This phenomenon is more evident when analyzing in detail the median and IQR values (Table 2), where a trend can be observed not reaching statistical significance (U = 69.5; *p* = 0.205).

**Figure 2:**
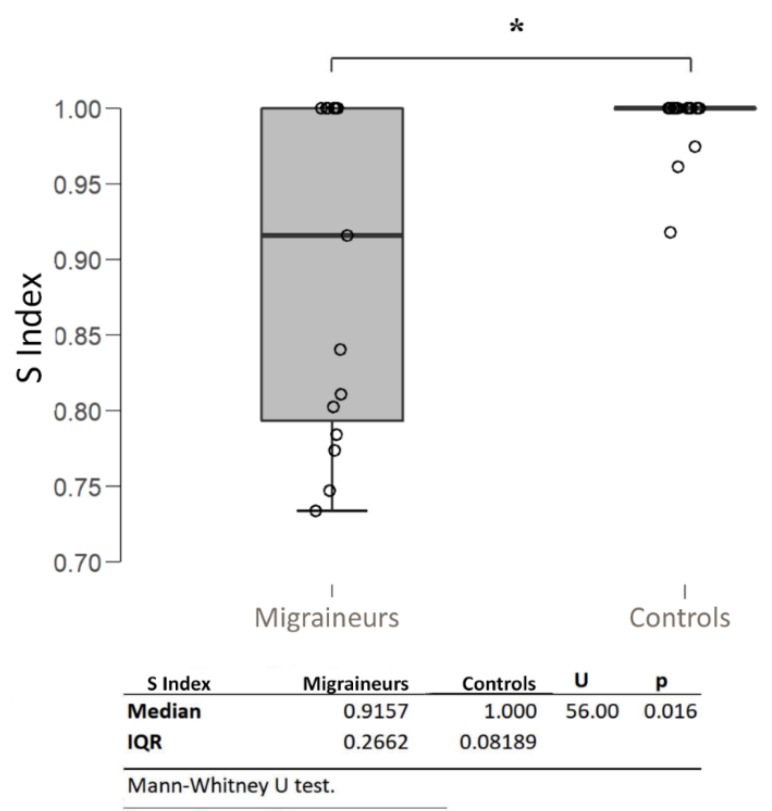
S index distribution among the study populations. Higher variability and lower tail values of the S index are registered in migraine patients confronted with controls. * *p* < 0.05.

Migraine patients showed lower values of S index compared to controls (U 59; *p* = 0.016) (Figure 2), being the minimum value of 0.73 for migraine patients and 0.91 for controls. The percentage of patients expressing supersaturation (S < 0.95) is 53%, while only 7% of controls demonstrate this characteristic (FET *p* = 0.0157).

**Figure 3:**
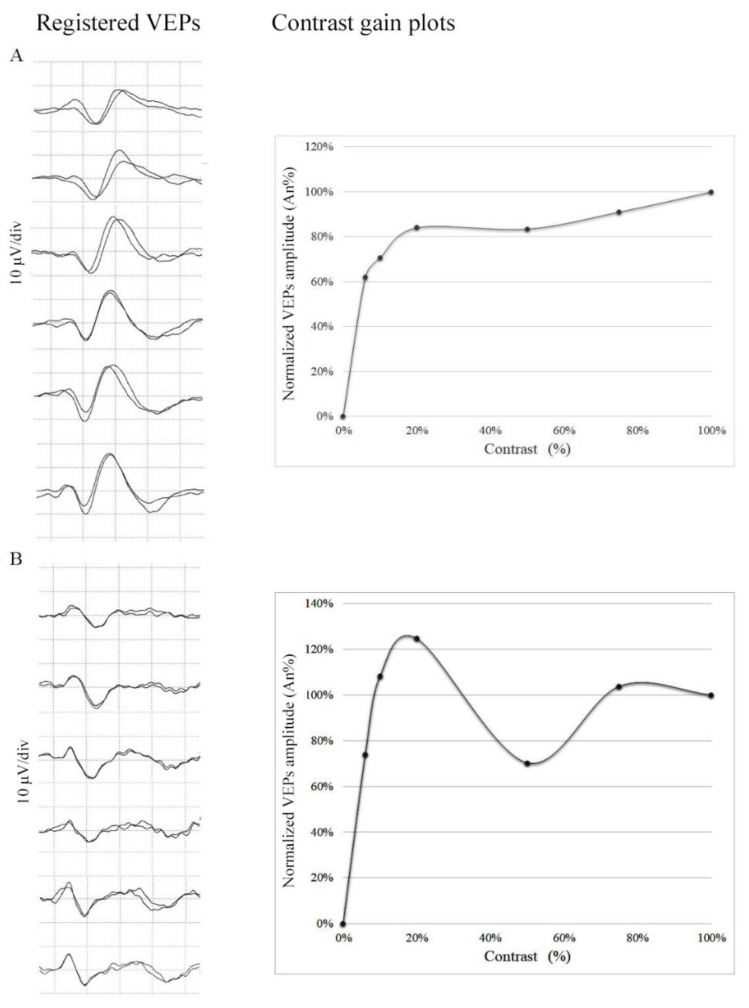
Graphic plotting of VEP amplitude to contrast gain: (**A**) control subject with normal saturation at maximum contrast (S = 1) and (**B**) a migraine patient with supersaturation (S = 0.80). The left column shows the signal registered for each subject at different contrasts (6%, 10%, 20%, 50%, 75%, and 100%) (50 ms horizontal divisions and 10 µV vertical divisions). The right column visualizes the plots of normalized amplitude (considering VEP amplitude registered at the contrast of 100%). The examples were chosen to visualize how different individuals could show signal amplitudes not comparable in terms of raw microvolts. The normalization is indeed necessary to compare the results avoiding misleading interindividual differences.

Examples of the recorded potentials for both groups have been represented with normalized amplitude graphs in Figure 3. The contrast gain of VEP approaches the maximum amplitude at 20% of contrast in controls while showing a paradoxical decrease in migraineurs with supersaturation.

The analysis of factors that could allow the differentiation or stratification of migraine patients based on the S index was made considering the anamnestic characteristics and the scores of administered questionnaires and scales.

The analysis of medians (Table 3) showed a clear reduction in patient quality of life, with slight mood deflection and moderate elevation of anxiety. The S index at T0 correlate significantly both with ASC-12 values (ρ_s_ = −0.736; *p* = 0.002) and the days to the next migraine attack (ρ_s_ = −0.915; *p* < 0.001).

The supersaturation phenomenon is enriched excluding from the analysis of patients not satisfying the intercritical period definition of VEP recording. The percentage of the remaining 12 patients showing supersaturation during intercritical period is 67%, and there is a significant difference comparing normalized amplitude at 20% of contrast (A_n20_%) between them and controls (U = 32; *p* = 0.011. Table 4). Hence, A_n20_% during the intercritical period can discriminate between patients and controls with optimal specificity (specificity = 1) and poor sensitivity (sensitivity = 0.42). 

The number of patients who completed the study at T1 was 8. The remaining 7 interrupted the treatment for drug-related side effects or did not tolerate the prescribed dose (100 mg per day). These patients discontinued the study. The percentage of drug-responsiveness (defined as ≥50% reduction in headache frequency) was of 75% in the T1 patients. The median headache frequency of these 8 patients at T0 was 9 days per month (IQR = 4), while at T1, it was 4 days per month (IQR = 4) with a significant reduction (W = 21; *p* = 0.036). The reduction in both HIT-6 (W = 28; *p* = 0.022) and MIDAS (W = 21; *p* = 0.036) scores is statistically significant, however, the disability grade was not affected notably. No change was measured regarding the other administered questionnaires (Table 5). 

**Figure 4:**
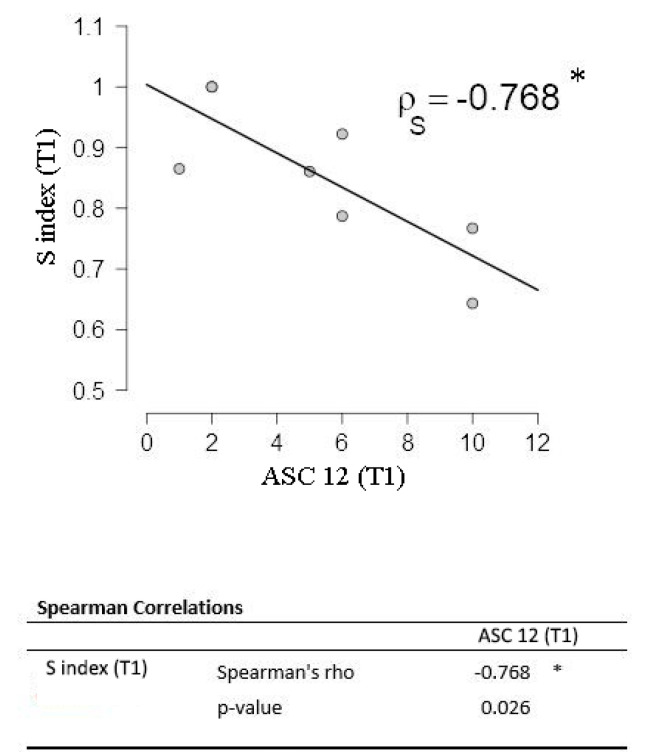
S index correlation with Allodynia Symptoms Check-list 12 (ASC-12) is confirmed at follow-up (T1). * *p* < 0.05.

The S index did not show differences before and after the treatment (W = 38; *p* = 0.80). The percentage of patients showing supersaturation at T1 is 62%, however, in this case, it was not possible to distinguish between critical and intercritical periods for the absence of information about the days following the second VEP recording. Moreover, further subanalysis could result in an insufficient sample size. The correlation between ASC-12 and S index was confirmed at T1 (ρ_s_ = −0.768; *p* = 0.026) (Figure 4).

## 4. Discussion

This study evidences a different visual cortex response to contrast gain assessment of VEP between migraine patients and controls. Specifically, the supersaturation, as a reduction in VEP amplitude at high contrast, is almost exclusively expressed in the migraineurs. Neuronal circuitry and morpho-functional aspects that could justify this habit are not well-defined considering the presented data, nor literature evidence. One of the possible explanations could rely on the hyperresponsiveness to the incremental contrast of the visual cortex in migraine, due to a positive feedback mechanism underlying disease predisposition [32]. This facilitation could ease the excitation of cortical neurons, registered through the augmented evoked potentials amplitude at lower contrast. The contribution of non-neuronal cells to synaptic excitability and the emerging role of neurovascular unit (NVU) in neurological diseases should also be considered [33,34]. This mechanism could link hyperresponsiveness to hyperexcitability. The hyperexcitability, however, could not be explained with reduced inhibitory systems (as for epilepsy), in fact, in this scenario, VEP amplitude should not saturate at high contrast [6,35]. The registered supersaturation is, on the contrary, a compensatory maladaptive phenomenon of increased cortical inhibition. A possible explanatory mechanism is the excessive lateral inhibition within the visual cortex [36]. However, the complexity of the non-linear properties of visual cortex responses could be explained by both cortical and subcortical pathways [23,37].

For instance, it was shown that relay cells from the lateral geniculate nucleus saturate their response at contrast ≥ 32%. The dysfunctional response of altered inhibition of these cells could lead to a feed-forward pathway for cortical hyperresponsiveness. The peculiar response of these cells, whose activation saturate with contrast gain, is fascinating but other cellular processes could influence the excitatory threshold and the response to visual stimuli [37]. Our data support the non-exclusively cortical genesis of hyperresponsiveness. Indeed, topiramate apparently did not interfere with supersaturation and its mechanism should reduce cortical excitability and retinal afference. A limitation of this study is the sample size; however, the results are consistent both at T0 and T1. The reduction in VEP amplitude registered with the S index in the 7% of controls, moreover, seems to have different characteristics, since it never overcomes the 10% and it is registered at high contrast (75%). This consideration justifies the high specificity of the electrophysiological findings.

The development of predictive biomarkers of specific drug responses remains an unmet need in the prophylactic treatment of migraine. However, the analysis did not achieve the discriminatory power to stratify migraine patients based on the electrophysiological findings, since scarce correlation has been noted with clinical characteristics or treatment response. Patients showed compromised quality of life, minor mood deflection and moderate anxiety while fatigue, even considering the variability among patients, did not seem a prominent symptom. It could be of interest to observe the scarce quality of sleep perceived among patients even with no daytime sleepiness and a normal chronotype. These data can represent the homogeneity of the selected patients with a moderate disease phenotype. Even if the clinical response to topiramate treatment has been registered, the reduced sample size could be partially responsible for the lack of predictivity of the S index. Quite the reverse could be stated about the dichotomous diagnostic variables (phonophobia and photophobia) since their intrinsic high prevalence in the sample could not allow a correlation with the S index. To overcome this limitation, it could be useful to use or design questionnaires that investigate the magnitude of the *-phobias* symptoms rather than their presence/absence. The sample was selected to be homogeneous and this bias could explain the incapability of the S index to differentiate patients considering their headache frequency. The years of illness, on the other hand, seem to be unrelated to the VEP response, indeed the S index could represent a marker of the disease that does not vary with time. 

The S index could be considered a biomarker of the migraine cycle, rather than a diagnostic/prognostic factor for therapy management of the single patient. Our data suggest that the S index strictly correlates with the days to the next headache attack. This aspect of the migraine cycle shows a surprising symmetry with the fMRI, where the Broadman areas 17–19 of the visual cortex demonstrated a peculiar pattern of activation related to the distance from the migraine attack [16]. The same cyclic pattern has been shown in sensorimotor and visual cortices by both electrophysiological and neuroimaging studies [2,3,11,36,38,39]. Functional neuroimaging or combined electrophysiological techniques could help to discriminate morpho-functional elements.

The sleep/wake pathophysiology instead does not influence the S index, even if it seems related to the migraine cyclic pattern presumably through the hypothalamic regulatory clock. 

The recording of an electrophysiological biomarker, such as the S index, related to the migraine cycle (i.e., the days to the next pain-phase), could be further characterized considering the intercritical quantification of other indicators of cortical hyperresponsiveness, through clinical assays (i.e., photophobia and phonophobia).

Another VEP study that considered the days separating the recording from migraine attack (both the last and next attack in this case) did not find a significant correlation [6]. However, it was performed with stimuli that elicited SS-VEP and patients were selected with higher headache frequency variability, although the group sizes are comparable. In our study, the exclusion of certain patients with very sporadic or high-frequency/chronic form of migraine could have highlighted the visual cortex hyperresponsiveness, indicated by the S index, and its relationship with the pain reoccurrence. This phenomenon could be observed almost exclusively in a very homogeneous sample. The hypothesis is that a common baseline (at least 3 days to the previous headache attack) and comparable frequencies allowed the alignment of the patients’ cyclic patterns of migraine. This configuration could not be achieved considering highly variable headache frequencies. 

Patients with high-frequency headache attacks (more than 10 per month) were excluded for the intrinsic difficulty to find the intercritical phase. A clear definition of migraine patterns and group homogeneity is mandatory to investigate the electrophysiological modifications of cortical activity. These data encourage validating experiments with larger samples and further studies that could observe the temporal modification of the S index. VEP analysis with contrast gain protocol, registered at various timepoints, of the same subject could remove interindividual variability and eventually corroborate or reconsider the hypothesis of the S index as a biomarker of the migraine cycle.

Nonetheless, the therapeutical response could help to understand this phenomenon and possibly furnish some hints about the underlying mechanisms, such as neurotransmitters involvement or central/peripheral sensitization [26,40,41]. On the other hand, it could be possible to obtain predictive information about drug-efficacy, advancing towards the precision medicine target [42].

Allodynia represents a clinical index of sensitization in migraineurs, and the strict correlation of ASC-12 and S index both at T0 and T1 could link the visual hyperresponsiveness to other cortical areas (e.g., somatosensory cortex) and the trigeminal system [43,44,45] with the unbalanced excitatory/inhibitory inputs being responsible for this clinical manifestation. In this scenario, the hyperresponsiveness is not limited to the visual cortex and the S index could be a biomarker of cortical sensitization in a single patient. ASC-12, in fact, represents the measure of allodynia during a severe migraine attack, and lower values of the S index, although mainly registered during the intercritical phase, it could predict worse critical symptoms.

## 5. Conclusions

This study confirms that VEP amplitude varies significantly in migraine patients compared to controls. In this experimental setting, supersaturation of VEP was considered. The results do not allow to distinguish the specific mechanism underlying the supersaturation, nor the involved anatomical structures. Nonetheless, topiramate therapy did not modulate the electrophysiological measurements suggesting a non-retinal nor cortical source of the supersaturation, with possible contribution of the lateral geniculate nucleus. The S index could be relevant as a biomarker for the migraine cycle and cortical hyperresponsiveness though more studies are needed. Further contributions could come from the registration of the S index at different timepoints during the migraine cycle and the susceptibility of the same index to different prophylactic therapies, also to collect predictive information about drug efficacy, advancing towards the precision medicine target. The choice of pattern-reversal stimulation and transient responses was proposed since it is available for a clinical development. If validated for clinical routine, a hypothetical 2 contrast measurement (20% and 100%) could be used to assess a simplified S index, reducing the time of registration.

## Figures and Tables

**Table 1:** Descriptive table comparing demographic information of patients and controls.

Variable	Controls	Migraineurs		
Number	13	15		
Male	4	4	*P* = 1 ^a^	
Female	9	11		
Age median (IQR)	30 (27)	34 (38)	*P* = 0.89 ^b^	

^a^ Fisher Exact Test (FET); ^b^ Mann–Whitney U test. IQR = interquartile range.

**Table 2:** The normalized amplitude at single contrast confronting migraineurs and controls.

	Normalized VEP Amplitude (A_n%_) Median (IQR)			
Contrast	Controls	Migraineurs	U	*P*
6%	53% (52)	51% (62)	95.50	0.945
10%	60% (70)	68% (99)	88.50	0.695
20%	69% (39)	83% (93)	69.50	0.205
50%	78% (46)	85% (38)	81.50	0.475
75%	91% (28)	94% (55)	81.50	0.475

Mann–Whitney U test. Values expressed in median and interquartile range (IQR), graphically plotted in Figure 1. No significant differences were found at single-contrast analysis.

**Table 3:** Correlation of S index and clinical features of migraine patients at baseline (T0).

Correlation		Median (IQR)	Spearman’s Rho		*p*
**S Index**	–	0.9157 (0.2662)	–		–
–	Frequency (days/months)	8 (5)	−0.315		0.252
*–*	Days to the next attack	3 (11)	−0.915		<0.001 ***
–	Years of disease	20 (38)	−0.030		0.915
–	Attack mean duration (h)	24 (64)	0.437		0.103
–	HIT-6	67 (27)	0.023		0.936
–	ASC-12	4 (11)	−0.736		0.002 **
–	ESS	5 (10)	0.229		0.411
–	PSQI	8 (9)	0.419		0.120
–	rMEQ	16 (12)	−0.281		0.310
–	FSS	32 (40)	0.000		1.000
–	GAD-7	13 (20)	−0.074		0.795
–	PHQ-9	12 (16)	−0.241		0.386
–	MIDAS	8 (44)	−0.463		0.082
–	VNS	7 (3)	0.138		0.623

The table report data expressed as median and interquartile range (IQR) and the significance of the correlation test (** *p* < 0.01, *** *p* < 0.001). Verbal Numeric Scale (VNS); Headache Impact Test (HIT-6); Migraine Disability Assessment (MIDAS); Allodynia Symptoms Check-list 12 (ASC-12); Fatigue Severity Scale (FSS); Generalized Anxiety disorder (GAD–7); Patient Health Questionnaire (PHQ–9); Epworth Sleepiness Scale (ESS); reduced Morningness-Eveningness Questionnaire (rMEQ); Pittsburgh Sleep Quality Index (PSQI).

**Table 4:** The normalized amplitude at single contrast confronting migraineurs (during the intercritical period) and controls.

	Normalized VEP Amplitude (A_n%_) Median (IQR)			
Contrast	Controls	Migraineurs (Intercritical)	U	*P*
6%	53% (52)	58.5% (59)	64.50	0.479
10%	60% (64)	69% (92)	58.00	0.288
20%	69% (39)	93% (76)	35.00	0.021 *
50%	78% (46)	86% (38)	61.50	0.384
75%	91% (28)	99% (55)	55.00	0.221

Mann–Whitney U test. Values expressed in median and interquartile range (IQR). Significant differences were showed at contrast of 20% (* *p* < 0.05).

**Table 5:** Comparative analysis of S index and data from clinical assessment, patient’s diary, and questionnaires between T0 and follow-up (T1).

	Median (IQR)			
	T0	T1	W	*p*
S index	0.83 (0.27)	0.86 (0.36)	16.000	0.800
Frequency (days/month)	9 (4)	4 (9)	21.000	0.036 *
VNS	7 (2)	7 (2)	1.500	1.000
Attack mean duration (h)	36 (64)	12 (64)	6.000	0.181
HIT-6	67.5 (27)	62.5 (51)	28.000	0.022 *
MIDAS	11 (44)	10 (40)	21.000	0.036 *
ESS	5 (10)	5 (14)	0.000	0.095
PSQI	7 (9)	7 (5)	3.000	1.000
rMEQ	15 (8)	16 (7)	0.000	0.371
ASC-12	5.5 (11)	5.5 (9)	9.000	0.786
GAD-7	7.5 (15)	6 (19)	17.000	0.670
PHQ-9	10 (16)	11 (17)	15.000	0.932
FSS	33.5 (40)	37 (34)	20.500	0.779

Wilcoxon signed-rank test. Significant values have been marked (* *p* < 0.05). Verbal Numeric Scale (VNS); Headache Impact Test (HIT-6); Migraine Disability Assessment (MIDAS); Allodynia Symptoms Check-list 12 (ASC-12); Fatigue Severity Scale (FSS); Generalized Anxiety disorder (GAD–7); Patient Health Questionnaire (PHQ–9); Epworth Sleepiness Scale (ESS); reduced Morningness-Eveningness Questionnaire (rMEQ); Pittsburgh Sleep Quality Index (PSQI).

## Data Availability

The data presented in this study are available on request from the corresponding author.
